# *In situ* vocal fold properties and pitch prediction by dynamic actuation of the songbird syrinx

**DOI:** 10.1038/s41598-017-11258-1

**Published:** 2017-09-12

**Authors:** Daniel N. Düring, Benjamin J. Knörlein, Coen P. H. Elemans

**Affiliations:** 1 Department of Biology, University of Southern Denmark, Odense, Denmark; 2 Center for Computation and Visualization, Brown University, Providence, RI USA; 3 Present Address: Institute of Neuroinformatics, ETH Zurich and University of Zurich, Zurich, Switzerland

## Abstract

The biomechanics of sound production forms an integral part of the neuromechanical control loop of avian vocal motor control. However, we critically lack quantification of basic biomechanical parameters describing the vocal organ, the syrinx, such as material properties of syringeal elements, forces and torques exerted on, and motion of the syringeal skeleton during song. Here, we present a novel marker-based 3D stereoscopic imaging technique to reconstruct 3D motion of servo-controlled actuation of syringeal muscle insertions sites *in vitro* and focus on two muscles controlling sound pitch. We furthermore combine kinematic analysis with force measurements to quantify elastic properties of sound producing medial labia (ML). The elastic modulus of the zebra finch ML is 18 kPa at 5% strain, which is comparable to elastic moduli of mammalian vocal folds. Additionally ML lengthening due to *musculus syringealis ventralis* (VS) shortening is intrinsically constraint at maximally 12% strain. Using these values we predict sound pitch to range from 350–800 Hz by VS modulation, corresponding well to previous observations. The presented methodology allows for quantification of syringeal skeleton motion and forces, acoustic effects of muscle recruitment, and calibration of computational birdsong models, enabling experimental access to the entire neuromechanical control loop of vocal motor control.

## Introduction

Vocal motor control involves at least three different motor systems - the respiratory system, vocal organ, and upper vocal tract^1–3^- that incorporate neural and mechanical feedback and thus form a closed control loop of which the biomechanics form an integral part^3, 4^. Birdsong has developed into a highly productive model system for vocal imitation learning^5–7^, and motor sequence learning^8^ and is also an excellent model to study vocal motor control because, in contrast to humans, we potentially have experimental access to the entire neuromechanical control loop. Additionally, roughly half of the 10.000 extant bird species are songbirds and their complex and elaborate song is considered to have played a major role in their evolutionary success^9^ warranting study of the underlying mechanisms of vocal control. In the last decade significant progress has been made towards unraveling central connectivity and neural mechanisms of the song system^7, 8^, and has seen the inception of neuromechanical models that include neural pathways and biomechanical syrinx models^10, 11^. However, we still have little quantitative insight in the biomechanics of avian sound production and control^2, 3, 12^. Critically we lack quantification of basic biomechanical parameters such as material properties of syringeal elements, forces and torques exerted on, and motion of the syringeal skeleton during song^3^. Such data are essential to (1) predict the behavioural (acoustic) outcome of neural signals, (2) investigate which physical mechanisms modulate acoustic parameters of the radiated sound waves, such as fundamental frequency and amplitude (3) what physiological relevant control range of skeletal motion is available to different species of birds and (4) develop and validate neuromechanical models that include realistic material properties, geometries and muscles. Here, we combine novel 3D stereomicroscopic techniques with force measurements to quantify deformation and *in situ* material properties of sound producing membranes by actuating muscle insertion sites in the zebra finch syrinx.

The well-studied physical mechanism of sound production in mammals is based on airflow-induced self-sustained oscillations of viscoelastic soft tissue translating mechanical energy into acoustic energy, also known as the myoelastic-aerodynamic (MEAD) theory^13, 14^. Several decades of studying MEAD based sound production in humans and other mammals has shown that fundamental frequency (*f*_0_)^15^ of vocal fold oscillations, which determines the *f*_0_ of the radiated sound, is set by the resonance properties of the vocal folds. These resonance properties in turn are predominantly determined by vocal fold length and morphology, such as collagen and elastin fibre composition and orientation of different vocal fold layers^16, 17^. Different layers of the vocal folds can exhibit different resonance properties and participate to varying degrees in oscillation, distinctly changing the *f*_0_ of oscillation^18, 19^. The complicated dynamic interactions between fluid, structure and acoustics make it difficult to predict real-time *f*_0_ during sound production, but surprisingly simple one-dimensional string models that estimate vocal fold resonance properties can provide reasonable estimates for the available *f*_0_ range in mammals^20–23^. Such models are based on only vocal fold length and elastic properties, where the elastic modulus (or Young’s Modulus, or spring constant) is a measure of an objects resistance to deformation, described as the ratio between stress (force per area) and strain (proportional deformation). The spring constant has been quantified for vocal fold tissues in several mammalian species including humans^24–26^, but not yet in any birds.

**Figure 1:**
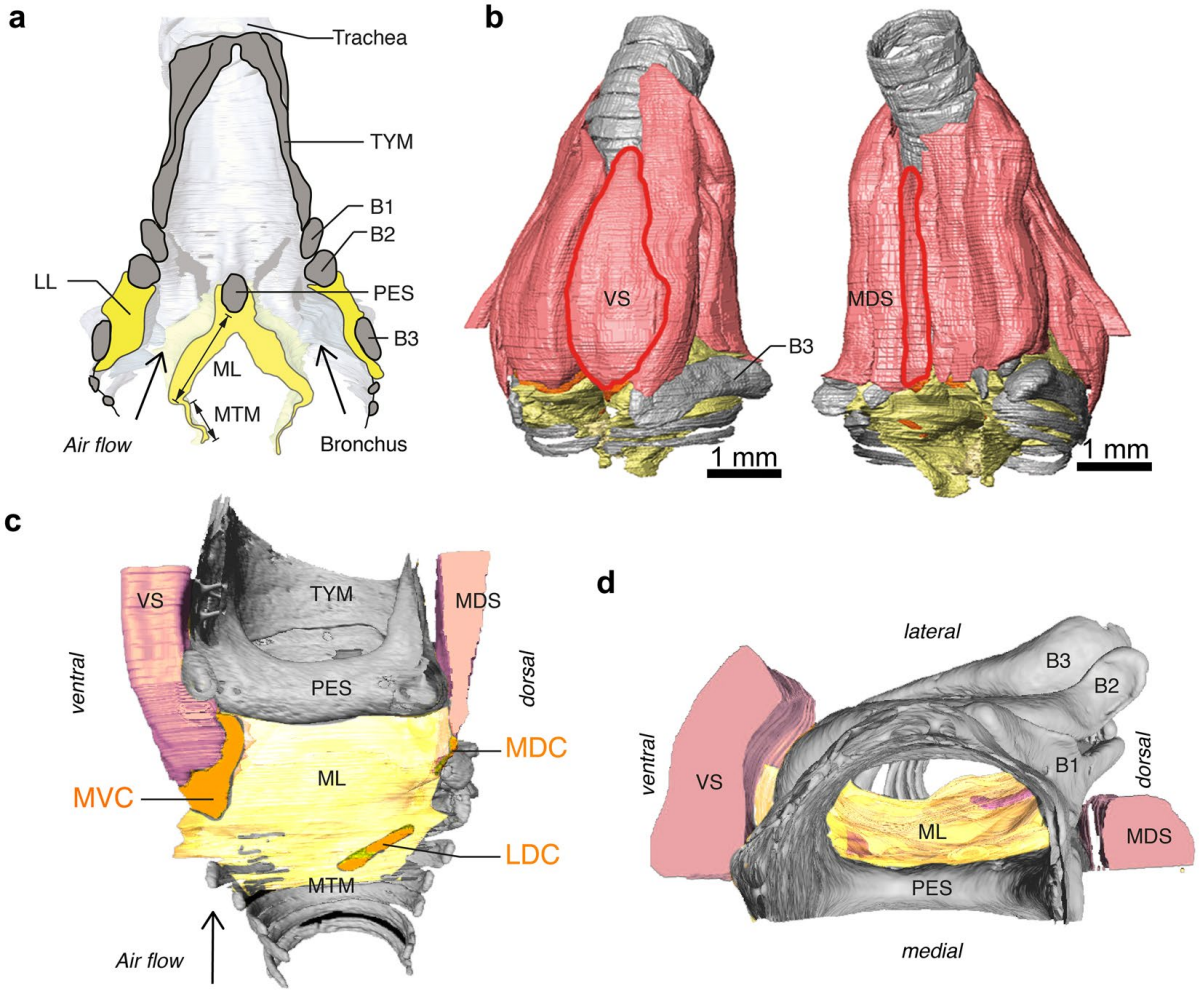
Syrinx morphology. (**A**) Schematic sagittal cross-section through the syrinx showing the syringeal skeleton (grey) with the paired soft tissue masses LL and MVM (yellow). Muscles are omitted for clarity. (**B**–**D**) Micro-computed tomography scan of the zebra finch syrinx (modified from ref. 30) showing bones (grey), cartilaginous pads (orange), muscles (pink) and sound producing MVM (yellow). (**B**) Ventral and dorsal view with the two muscles (red outlines) that directly attach on cartilaginous pads embedded in the medial labium, VS and MDS. (**C**) Medial view on the MVM with only soft tissues of right bronchus visible and left bronchus removed for clarity. The tympanum is the bony cylinder that is formed of fused tracheal rings. The pessulus is an ossified medial dorso-ventral bridge of the tympanum dividing the two bronchi. (**D**) Top view of panel C. For a detailed description of the zebra finch syrinx including 3D interactive PDF figures see ref. 30. Abbreviations: B1-3, bronchial bones 1-3; LDC, latero-dorsal cartilage; LL, lateral labium; MDC, medio-dorsal cartilage; MDS, *musculus syringealis dorsalis medialis*; ML, medial labium; MVC, medio-ventral cartilage; MVM, medial vibratory mass; PES, pessulus; TYMP, tympanum; VS, *musculus syringealis ventralis*.

We recently presented an *ex vivo* paradigm to study avian sound production and established that birds, like mammals, despite their different evolutionary origin also employ the MEAD mechanisms for sound production^27^. The morphology of the avian vocal organ, the syrinx, is very different from the mammalian larynx. In songbirds the syrinx has two sound sources – one in each bronchus – that can oscillate independently^28, 29^. Each source consists of two oscillating tissue masses: on the lateral side the lateral labium (LL) located inside half-ring shaped bronchial bone B3, and the medial vibratory mass (MVM) suspended on the medial side (Fig. 1; See ref. 30 for a detailed description of the zebra finch syrinx including interactive 3D PDF figures). During *ex vivo* sound production, large amplitude caudo-cranial oscillations of the MVM open and close the bronchial lumen^27^. In zebra finches both syringeal closure and opening is associated with sound excitation of the upper vocal tract^27^. The mediolateral thickness of the zebra finch MVM varies along the caudiocranial axis^2, 30, 31^ dividing it into two distinct parts, a thicker medial labium (ML) and the membranous medial tympaniform membrane (MTM) (Fig. 1A). Predominantly the thicker ML seems to set MVM resonance properties and hence sound *f*_0_, because both rupture^32^ and damping^31^ of the thin MTM leave the radiated sound *f*_0_ relatively unaffected. The role of the ipsilateral LL is yet unclear as it is difficult to observe during sound production. The LL has no direct muscle attachments and it thus seems unlikely that LL length or tension is under motor control. Therefore we proposed that the LL phase-locks to the oscillation frequency set by the ML^30^.

In mammals the tension and length of vocal folds is set by the morphology of surrounding cartilages and controlled by cricothyroid and thyroarytenoid muscles^33, 34^. The zebra finch syrinx contains eight pairs of muscles, which act on the skeletal framework^30^. These muscles all insert on one end to the bony cylinder that makes up the undivided part of the songbird syrinx, the tympanum (TYM in Fig. 1) and their shortening thus actuates parts of the syringeal skeleton relative to the tympanum. Two of these muscles, *musculus syringealis ventralis* (VS) and *musculus syringealis dorsalis medialis* (MDS), attach to cartilaginous pads that are embedded in the ML and could therefore directly modulate ML length and tension (Fig. 1B–D). The VS inserts on the medio-ventral cartilage (MVC), which is a flexible cartilaginous continuation of ossified bronchial bone B2 (Fig. 1; see ref. 30 for detailed descriptions of muscle insertion sites). The MDS inserts on the medio-dorsal cartilage (MDC) that is not directly connected to any ossified syringeal structures, and is embedded in the MTM. Indeed shortening of both VS and MDS muscles has been shown to affect *f*_0_: direct electrical stimulation of VS in zebra finches *ex vivo* ^27^ and Bengalese finches *in vivo* during song^35^ resulted in *f*_0_ increase of sound. These experiments supported earlier electromyographic (EMG) measurements of VS activity correlated to *f*_0_ in syllables of brown thrashers^36^ and to *f*_0_ in low frequency syllables in zebra finches^37^. However it should be noted that in Bengalese finches the EMG of VS activity correlated next to *f*_0_ also to other acoustic parameters such as amplitude and entropy, which strongly suggested that these correlations were context specific^35^. To our knowledge no MDS specific EMG data during song exists, but pulling MDS beyond its natural range caused *f*_0_ increase^31^.

In contrast to mammalian vocal folds, very few studies have quantified material properties relevant to sound production in the syrinx. The small size and anatomy of the zebra finch ML make it difficult to quantify its elastic properties by isolating the tissue in a uniaxial stretch apparatus as commonly used in mammalian larynx studies^22, 26, 38, 39^. A previous study^31^ quantified resonance properties of the MVM in zebra finches and canaries using laser Doppler vibrometry at fixed static pressures by acoustic excitation of the membranes. However, it remains unknown how the elastic properties and length of the ML changes as a function of muscle shortening. In mammals VF strain range is reported to go up to 45% in dogs^40^ and up to 28% in humans^41^ during vocalization, but in birds it is unknown to what extent VS and MDS shortening modulates ML length and what the natural and available range of ML length modulation is.

Here, we developed novel stereomicroscopic high-speed imaging techniques to allow for 3D spatiotemporal reconstruction of strain-fields within MVM during *in vitro* modulation akin to muscle actuation. By systematically actuating the insertion sites of and quantifying the necessary modulation forces normally exerted by the two muscles that directly act on the ML, namely VS and MDS we measured the elastic modulus of the ML *in situ*. We show that the elastic modulus of ML is in the same range as mammalian vocal folds, demonstrating another parallel in MEAD systems, and that a simple resonance string model can predict the range of *f*_0_. This presented methodology opens up new possibilities for quantifying syringeal skeleton motion and forces *in vitro* and *ex vivo* (perfused whole organ), to quantify the acoustic effects of muscle recruitment, and calibrate and test biophysical models of sound production.

## Methods

### Subjects

19 Male zebra finches (*Taeniopygia guttata Vieillot*) were euthanized by Isoflurane (Baxter, Lillerød, Denmark) overdose. Vocal tracts were dissected out and submerged in oxygenated Ringer’s solution on ice^42^. All experiments were conducted in accordance with the Danish law concerning animal experiments and protocols were approved by the Danish Animal Experiments Inspectorate.

### *In situ* MVM actuation

**Figure 2:**
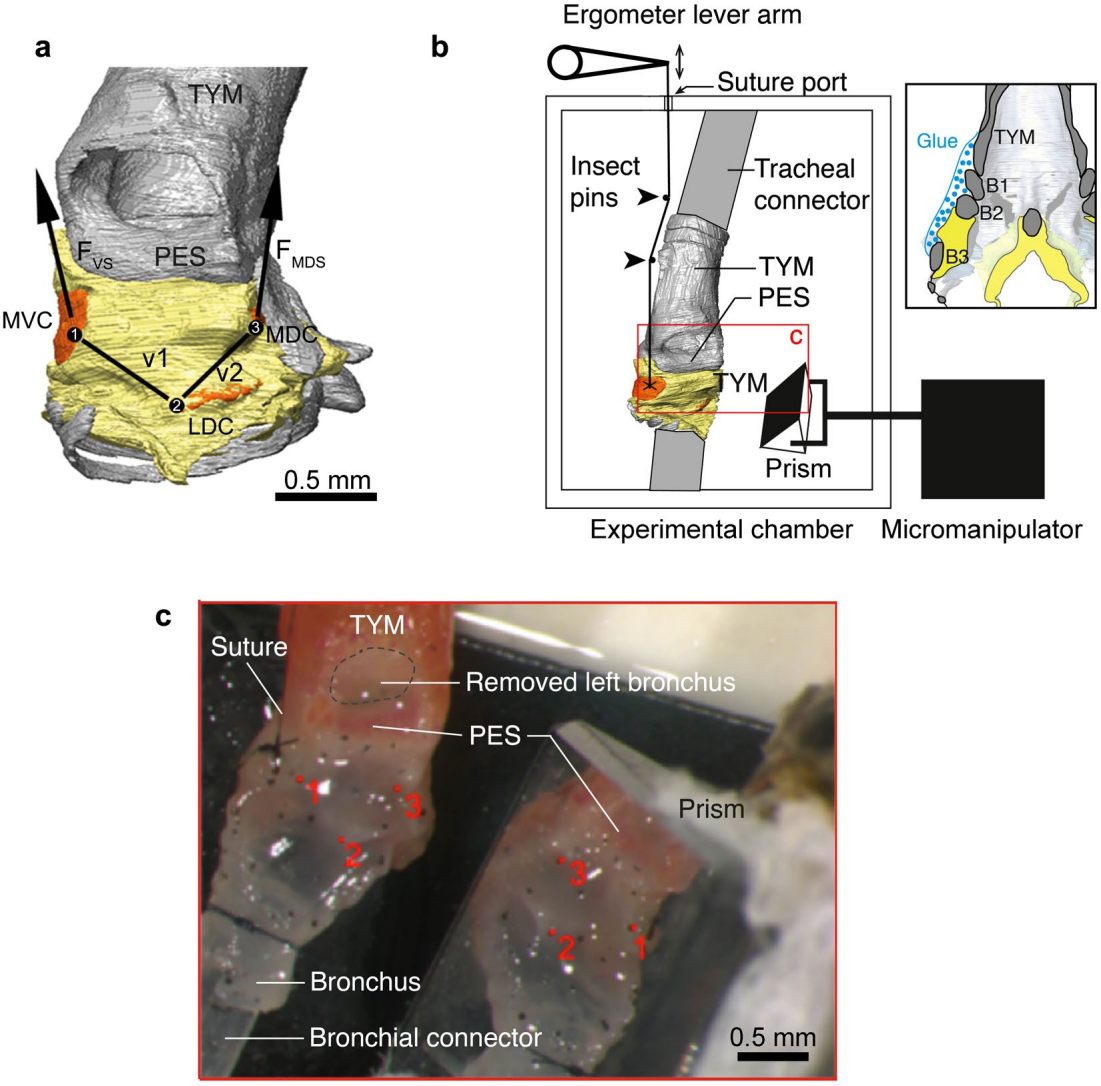
Experimental set-up. (**A**) Sideview of the right MVM (yellow) in relation to the syringeal skeleton (grey) with the left bronchus removed for clarity. Carbon markers (black dots labelled 1–3) were placed on the MVM to quantify its deformation. The lines of action of the force vectors the VS and MDS muscles exert are indicated (F_vs_ and F_MDS_). (**B**) Schematic drawing of the experimental set-up. The syrinx is fixed in the experimental chamber to a tracheal connector, consisting of rigid polyethylene tubing, and bronchial connector, consisting of silicon tubing. The lever arm of the ergometer is placed outside the chamber and connected to the cartilage (here MVC) of the syrinx through a single 10-0 suture that is threaded through a small hole in the chamber (suture port). A 5 mm right angle prism is positioned with a micromanipulator to view the markers from two different angles. The red lined box indicates view in panel C. The inset (right) indicates where B1-3 were glued to the tympanum (blue) to fix them in the same coordinate system. (**C**) Top (left) and mirrored side view (right) of the syrinx with markers as imaged through the microscope. The left bronchus is removed to make the right MVM visible. Carbon micro-sphere markers 1–3 (red dots) represent the ML landmarks (see text). Abbreviations as in Fig. 1.

In mammals, elastic properties of the vocal folds are typically measured in a uniaxial stretch apparatus *in vitro* ^22, 26, 38^. However, uniaxial test require isolated strips of material that are longer than wide in the fibre direction (about 10:1 length-width ratio) to prevent unwanted edge and clamping effects. The small size (<1 mm) and geometry of the zebra finch ML make isolation of such small strips experimentally challenging for measuring elastic properties of the oscillating tissue in an uniaxial stretch apparatus. Therefore we decided to determine elastic properties by actuating muscle insertion location *in situ* (Fig. 2). This approach will provide the functional range of strain modulation set by the muscles and also provide the required forces needed to actuate the syringeal skeleton.

The syringeal muscles of the right side were carefully removed allowing access to their insertion sites^30^. All dissections were done in oxygenated Ringers solution on ice^30^. Here we focus on VS and MDS, because they both directly insert on cartilaginous pads in the ML and are therefore in positions to modulate ML length. A 10–0 uni-filament suture thread was carefully attached through the MVC and MDC cartilaginous pads while the syrinx was still submerged in Ringers solution.

Because all syringeal muscle shortening actuates parts of the syringeal skeleton relative to the tympanum, we aimed to fix the tympanum in the coordinate system of the entire setup and quantify motion of the MVC and MDC relative to the tympanum. The positions of bronchial bones B1-B3 are precisely controlled by five other intrinsic syringeal muscles, but we currently lack quantitative knowledge on their position during song *in vivo*. Therefore we decided to prevent motion of the bronchial bones B1-B3 during actuation of MVC and MDC and fixed them to the tympanum by applying small amounts of tissue adhesive (3 M, Saint Paul, Minnesota, USA) directly onto bronchial bones B1-3 and tympanum with a glass capillary (Fig. 2B). The syrinx was then placed in an experimental chamber described in detail in Elemans *et al*.^27^. After mounting the tympanum was tightly secured to the tracheal connector, consisting of a rigid polyethylene tube, and the abovementioned fixation prevented motion of bones B1-3 relative to the tympanum upon hardening of the tissue adhesive. As such we ensured that the entire syrinx did not move and remained in the same spatial coordinate system as the entire setup. To actuate VS and MDS muscle insertion points, we connected the loose suture end to a small titanium hook attached to the lever arm of an ergometer (Model 300 C, Aurora Scientific, Ontario, Canada), which measured displacement and force at the tip of the lever arm (displacement and force resolution 1 µm and 0.3 mN, respectively). As the ergometer was placed outside of the experimental chamber containing the syrinx, 0.25 mm insect pins (Austerlitz, Czech Republic) on a silicon pad were used to align the suture thread in line with the muscles line of action and the lever arm.

We determined the force required to stretch the ML in a series of experimental runs, assuming serial elasticity in the displacement chain consisting of metal hook, suture, MVC/MDC cartilage and ML. First, we measured the inertial forces required to move the assembly without the suture connected to the MVC/MDC by applying 20 cycles of a sinusoidal displacement signal at 1 Hz. Because dynamical tensile tests on mammalian vocal folds are conducted at 1 Hz^21, 22, 25, 26^, we used the same settings to allow for direct comparison of our results. Next, we connected the suture to the MVC/MDC by flattening its end and suturing it on the MVC/MDC using a curved needle suture and repeated the same run (termed *intact cycles*). Finally, we carefully detached the ML from MVC or MDC using sharp tip micro scissors and repeated another run (*detached cycles*). The force required to displace the ML was estimated by subtracting the force signals of the *detached cycles* from the *intact cycles*. Due to the destructive final stage of the experiment, we could only modulate MVC or MDC per individual. To highlight the muscle that actuates the cartilage, MVC modulation experiments are referred to as *VS actuation run* and MDC modulation experiments as *MDS actuation run* hereafter.

Previous syringeal muscle stimulation experiments showed that syringeal muscles shorten maximally ~10% *in situ* (10% in ring dove *musculus tracheolateralis* ^42^ and 12% for zebra finch VS^35^). To provide a larger range of shortening, we set the displacement amplitude of the actuation runs to 20% of the *in situ* resting length of VS or MDS for each experiment. Muscle resting length were measured with callipers and ranged from 3.5 mm to 4.3 mm for VS and from 3.0 mm to 4.0 mm for MDS. The displacement of the ergometer arm was adjusted prior to each run so that ML was not stretched visibly. During runs, the ergometer arm was then moved in a sinusoidal fashion where half the cycle moved the arm away from the preparation by 20% and back to the zero-point, which stretched and released the ML. The other half of the cycle moved the ergometer arm towards the preparation to ensure relaxation of ML in between cycles.

Force and displacement signals were filtered with a custom build standalone 3rd-order low - pass Butterworth filter (10 kHz) and digitized at 25 kHz (NI-DAQ-6259, 16 bits, National Instruments, Austin, Texas, US). All data analysis software was developed in Matlab (MathWorks, Natick, Massachusetts, United States) unless stated otherwise. To calculate the force required to displace the ML we first aligned the intact run and detached run by cross-correlating the displacement signals and subsequently subtracted the force signals. To allow for tissue preconditioning^43^, we omitted the first 5 cycles of each run in further analysis.

### 3D reconstruction of MVM deformation

In human vocal fold stretch experiments, the displacement signal of the ergometer lever did not reliably represent the stretching performed in the labial tissue measured optically^39^, leading the authors to conclude that uniaxial stretch experiments might underestimate elastic moduli by a factor of approximately five. To avoid such errors we developed an optical stereo-imaging method to accurately measure ML strain *in situ*.

**Figure 3:**
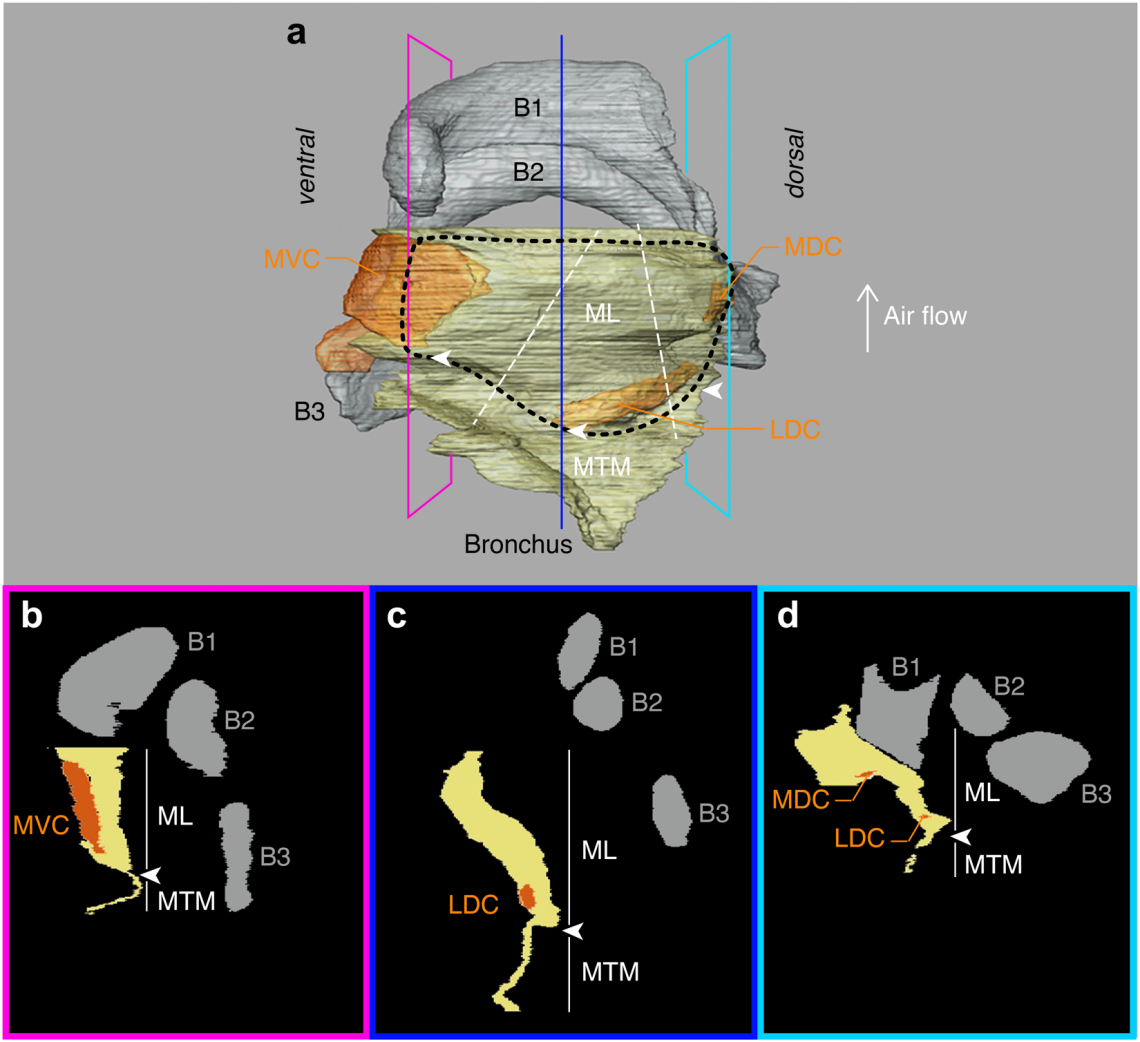
3D MVM morphology and tissue mass distribution. (**A**) Medio-lateral view of the zebra finch MVM (yellow) including cartilaginous pads (orange) and syringeal bones B1-B3 (grey). The thicker part that is referred to as the medial labium (ML) is outlined (black dotted line) based on sagittal sections in (**B**–**D**) as indicated by the pink, blue and cyan outlines. The MVM is thicker in the roughly triangular shape between MVC, LDC and MDC cartilages embedded in the MVM. The slanted white dashed lines indicate where the cross-sectional areas of the ML were measured (see methods). (**B**–**D**) Sagittal sections at locations shown in Panel A. The white arrowheads indicate the sudden narrowing that defines the border between ML and MTM. Abbreviations as in Fig. 1.

To allow 3D reconstruction of MVM deformation, we placed carbon microsphere markers (<80 µm diameter) on minimally 3 and up to 15 morphological landmark positions. The MVM was previously divided into ML and MTM based on relative thickness or mass. Tissue resonance properties along the midline of the MVM showed that the ML is most dominant in setting MVM’s resonance properties^31^. We identified the location of the largest tissue mass by analysing the MTM thickness in virtual cross sections of µCT data^30^ (Fig. 3). These data indicated that the MTM is thickest between the MVC, MDC and LDC and we confirmed this observation by visual observations in all fresh tissue preparations. Thus we can safely assume that the largest mass is also suspended between MVC, MDC and LDC and therefore we used these three conspicuous landmark marker positions to define the ML: namely the lateral edge of MVC, the caudal edge of MDC and the caudal-medial edge of LDC (Figs 1 and 2).

The MVM was imaged during the *intact cycles* with a high-speed camera (MotionPro-X4, 12 bit CMOS sensor, Photron, Tokyo, Japan) at a frame rate of 200 Hz mounted on a Leica M165-FC stereoscope (Leica Microsystems, Wetzlar, Germany). Images were synchronized to force and displacement data with an accuracy of <21 µs. Stereoscopic views of the syrinx were acquired by placing a 5 × 5 × 5 mm 45° angled aluminium surface-coated prism (Thorlabs, Newton, New Jersey, US) adjacent to the syrinx that could be fine positioned with a micromanipulator (model MM3, Narishige, Japan)(Fig. 2B,C).

For calibration of the projections, as well as tracking and reconstruction of markers we modified software originally developed for stereoscopic x-ray images, XMALab^44^, to light microscopy. To compute projections XMALab uses a two-stage calibration procedure. First, each projection was independently calibrated by waving a planar calibration grid (grid size of 400 µm^2^) through the 3D space where the syrinx was mounted, so-called checkerboard calibration. Second, the stereo camera setup was further refined using a non-linear optimization that minimized reprojection errors of the grid points while maintaining the same translation and rotation between both projection centres for all frames^44^. During calibration we filmed both projections for 30 seconds at a frame rate of 100 Hz and selected 60 images of the sequence. Calibrations were performed before and after each experiment. All experiments were performed at 21.5 ± 1.5 °C. At higher temperatures mirror fogging would prevent stereoscopic image acquisition. Due to the complex surgical procedures and high image quality requirement for 3D reconstruction, we performed several runs until all criteria were met and used the final run for analysis. After rejection of preparations based on poor calibration quality, we had an overall success rate of 37%, resulting in four specimens for VS and three for MDS modulation experiments.

Markers were tracked semi-automatically for each projection and their 2D locations were refined to subpixel accuracy in XMALab by fitting a polynomial with Gaussian weight to the image intensity function. The 3D locations of markers were then reconstructed using linear triangulation and the vectors between marker 1 and marker 2, and between marker 2 and 3 were named vector **v**1 and **v**2, respectively (Fig. 2A). For VS actuation runs we assume **F**_VS_ = **F**_**v**1_ and for MDS actuation runs we assume **F**_MDS_ = **F**_**v**2_ (Fig. 2A). Due to the non-planar and curved nature of the syrinx our stereoscopic setup did not allow for exact quantification of the angles between the muscles line of actions and **v**1 and **v**2 (Fig. 3). We estimate these angles to be below 30 deg in which case **F**_VS_ and **F**_MDS_ are maximally 14% lower.

### Elastic modulus

Strain between the markers was defined as:

1 $$\varepsilon =\frac{{\rm{\Delta }}L}{{L}_{0}},$$

where L_0_ is resting length in mm and ∆L change of length in mm. Stress was calculated as:

2 $$\sigma =\frac{F}{{A}_{0}},$$

where *F* is force in N and *A*_0_ is cross-sectional area in m^2^. Because the volume of nearly incompressible materials under stretch usually remains constant, the cross-sectional area has to change when length changes are imposed. This becomes important when calculating stress, i.e. the ratio of force to cross-sectional area. As the *in situ* nature of the experiment prevented measurements of the cross-sectional area, here the engineering definition of stress is used, which defines stress as the ratio of force to initial cross-sectional area. Because we could not directly measure initial cross-sectional area perpendicular to the strain vector in each preparation we instead measured this area in a virtual cross section of a 3D reconstruction (Fig. 3A) based on previous micro computed tomography scans^30^. The cross sectional area of the ML perpendicular to both of the two strain directions measured 0.02 mm^2^.

We fitted an exponential model to the measured stress-strain curves:

3 $${\rm{\sigma }}=A\,{e}^{{\rm{B}}{\rm{\varepsilon }}},$$

where A and B are constants. In mammalian studies the stress-strain response of vocal fold tissue is commonly differentiated into two parts, a low strain region and a high strain region. The low strain region is approximated with a linear model and the high strain region with an exponential model^20, 45^. The term Young’s Modulus is by definition only valid for linear elastic solid materials^46^. As there was no *a priori* reason to assume linear elasticity we fitted the stress-strain curve over the full strain range with an exponential model. While many mammalian studies calculate Young’s Modulus from a linear regression in the linear low strain region^23, 26^ we measured the tangent modulus as the derivative of the stress-strain curve over the entire range and will use the term *elastic modulus*.

**Figure 4:**
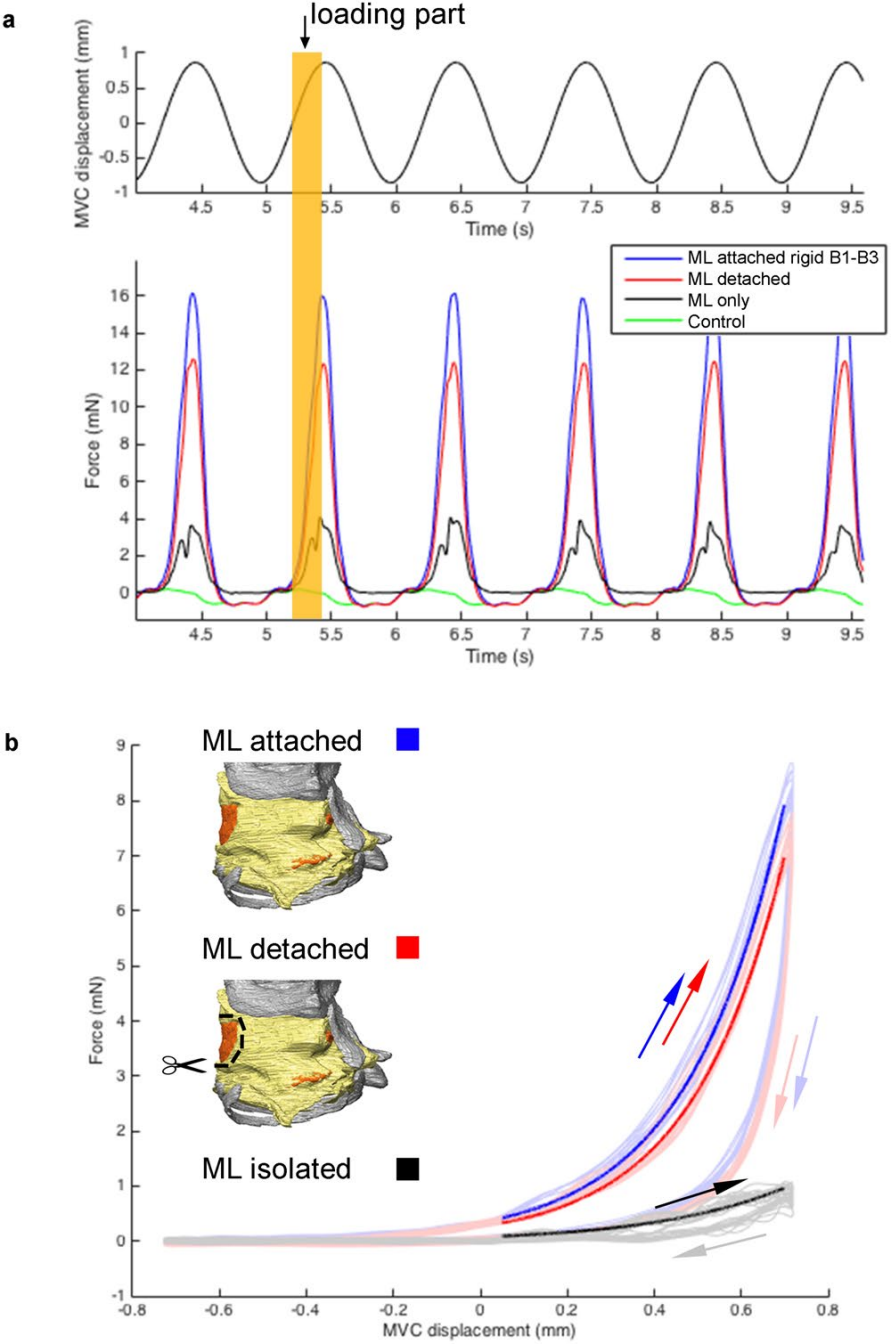
Typical raw MVC displacement and force data during a VS run. (**A**) Six cycles of MVC displacement (top) and resulting force (bottom) over time for the different experimental conditions; *fixed cycles* (blue lines), *detached cycles* (red), the difference force (black), and inertial forces of the setup (green) (see Methods). Each cycle starts with a positive displacement (defined as towards the motor, i.e. muscle shortening), pulling the MVC until peak displacement (here 0.86 mm) and back to resting position. The orange shaded area highlights the loading part of one cycle. (**B**) Force plotted against displacement resulting in work loops. Shown are 10 consecutive cycles (faded coloured lines) and the fitted loading curves (thick lines) for the three conditions. The loops run clockwise with the upper part of each curve representing the stretching or loading phase (solid arrows) and the lower part the unloading phase (faded arrows).

Elastic modulus *E* was calculated as:

4 $$E=\frac{\sigma }{\varepsilon },$$

where σ is stress and ε is strain. To calculate average ML stress, the loading parts of the force-displacement signals for intact and detached runs were first averaged over 15 cycles and subsequently subtracted. As the resulting forces were very low and small variations from the intact and detached cycles would show up amplified in the ML length signal shape, we fitted an exponential curve through the loading section of the ML signal (Fig. 4).

### Fundamental frequency estimates by string model

Due to the requirement to accurately position the mirror inside the chamber, we could not induce sound production as in ref. 27 while actuation muscle insertion sites. Therefore we predicted fundamental frequency using a linear string model:

5 $${f}_{0}=\frac{1}{2L}\sqrt{\frac{\sigma }{\rho }},$$

where L is length, σ is stress and ρ is density of the ML. As the small size and inaccessibility of the ML prevents accurate density measurements we use the value that is commonly used in mammalian vocal fold studies ρ = 1020 kg m^−3^ (ref. 22).

### Statistics

All data are presented as means ± 1 S.D.

### Data Availability

The datasets generated during and/or analysed during the current study are available from the corresponding author on reasonable request.

## Results

### Forces and deformation of the Medial Labium

We measured forces required to stretch the ML in the direction of the two muscles that can affect the *f*_0_ of sound - the VS and MDS - by cyclically displacing their respective muscle attachment site in two steps (Fig. 4). First, we displaced the intact system *in situ*. Second we detached the MVM (red line) and then displaced the muscle attachment site in order to calculate the force difference that represents the force required to stretch the ML alone. We obtained work loops, whose shape is dependent on the inertial and elastic characteristics of the system by plotting force against displacement (Fig. 4B). Loops rotate clockwise with the upper leg of the curve representing the stretching, or loading phase and the lower leg representing the release, or unloading phase. The area under the loading curve is the work required to stretch the tissue in the loading phase and is higher than the work returned in the unloading part. The area enclosed by the curve thus represents the (negative) work dissipated during each cycle. This viscoelastic property is commonly observed in soft tissue and demonstrates the hysteresis of vocal fold tissue^47^.

**Figure 5:**
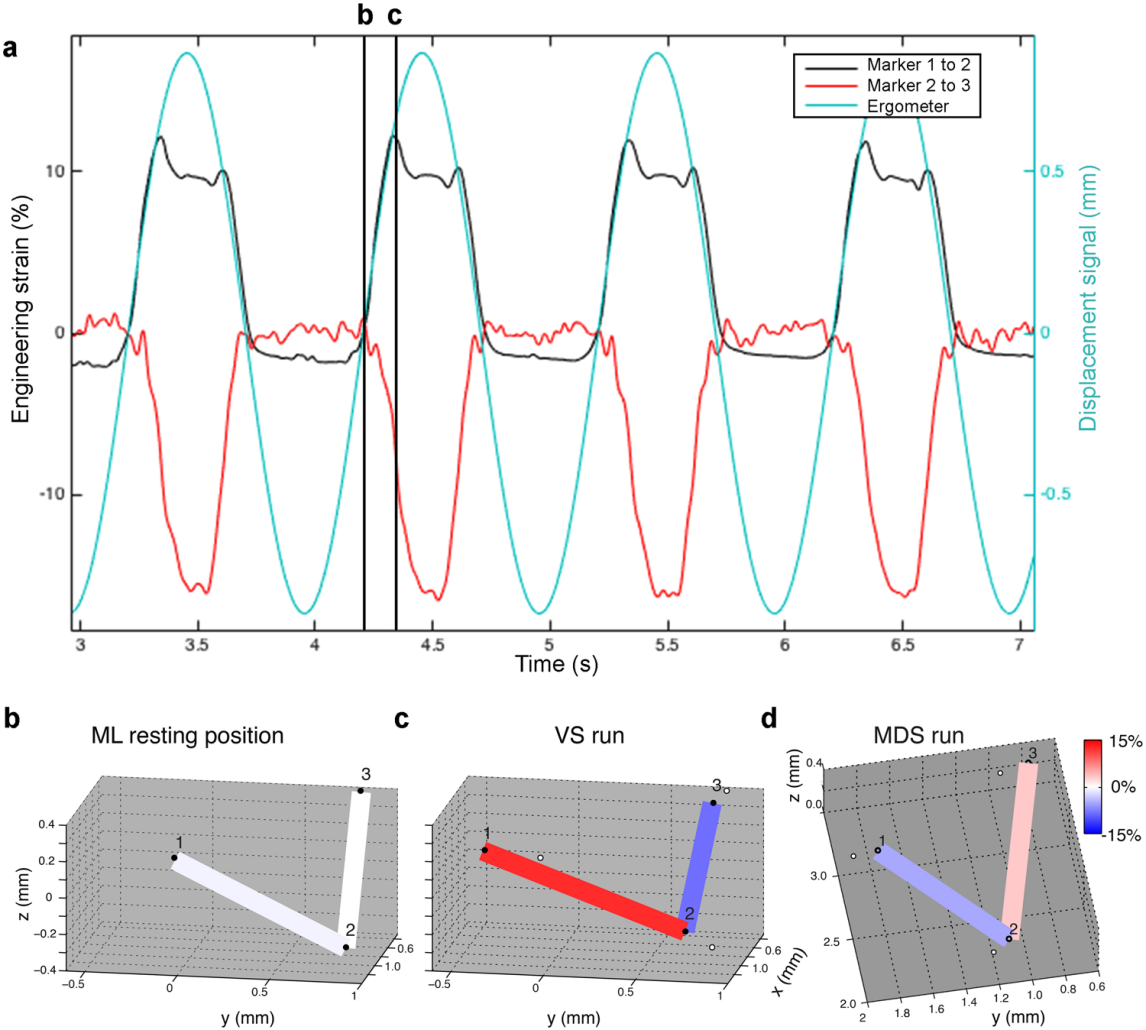
Stress-strain relationships for VS and MDS runs based on 3D stereoscopic marker tracking. (**A**) Inter-marker distances as engineering strain (**v1**, black; **v2**, red traces; left y-axis) and the displacement signal (light blue; right y-axis) during four successive cycles in a VS actuation run. At ~10% tissue strain, further displacement of MVC does not result in an increase in tissue strain. (**B**–**D**) Marker positions (black dots) at (**B**) resting position of ML, (**C**) maximal strain of ML during VS actuation run and (**D**) maximal strain during MDS actuation run. The strain increase (red) in the direction of the muscle line of action (VS in **C** and MDS in **D**) is accompanied by a strain decrease (blue) in the perpendicular direction. The white dots in panel **C** indicate the resting position of the markers (panel **B**). The exact orientation of the x,y and z axes and reference point (0,0,0) are defined by the position of the calibration wand during the calibration procedure. Please see Supplementary Videos S1 and S2 online for animations of the strain changes in ML during a VS and MDS run.

Because uniaxial human vocal fold tissue experiments have shown that applied length changes do not need to relate to vocal fold deformation^39^, we did not rely on ergometer displacement signals, but optically quantified 3D MVM deformation *in situ*. This technique allowed us to visualize the spatiotemporal position of ML landmark positions and calculate the engineering strain between these landmarks (Fig. 5). The resting length of ML between marker 1 and 2 (**v1**) measured 1.40 ± 0.30 mm and between marker 2 and 3 (**v2**) 1.24 ± 0.17 mm. Our data show that the applied cyclical strain at the muscle insertion sites does not reflect strain in the ML of zebra finches during the entire loading-unloading cycle under stretch conditions. ML strain increases with ergometer displacement signal during VS actuation runs (Fig. 5A), but only up engineering strain of 11.9 ± 7.9%, where **v1** strain reaches a plateau. A further increase in MVC displacement, does not lead to **v1** strain increase, but instead translates the entire ML. We observe the same phenomenon during MDS actuation runs, where the maximum strain plateau of **v2** is at 3.9 ± 4.2%. Using the displacement signal of the ergometer would thus result in erroneous stress-strain relationships above these plateau values and highlight the importance of optically measuring strain *in situ*.

Additionally we find that strain increase in line with the actuated muscle is accompanied by a strain decrease in the direction of the other actuation point. During VS actuation runs, positive MVC displacement (i.e., VS shortening) leads to ML strain increase in the direction parallel with the applied force (**v** **1**) and decrease in the direction perpendicular to the applied force (**v** **2**) (Fig. 5A,C; Supplementary Videos S1 and S2). During MDS actuation runs, positive MDC displacement (MDS shortening) leads to ML strain increase in the direction parallel with the applied force (**v** **2**) and decrease in the direction perpendicular to the applied force (**v** **1**) (Fig. 5B,D).

### Elastic modulus of the ML

**Figure 6:**
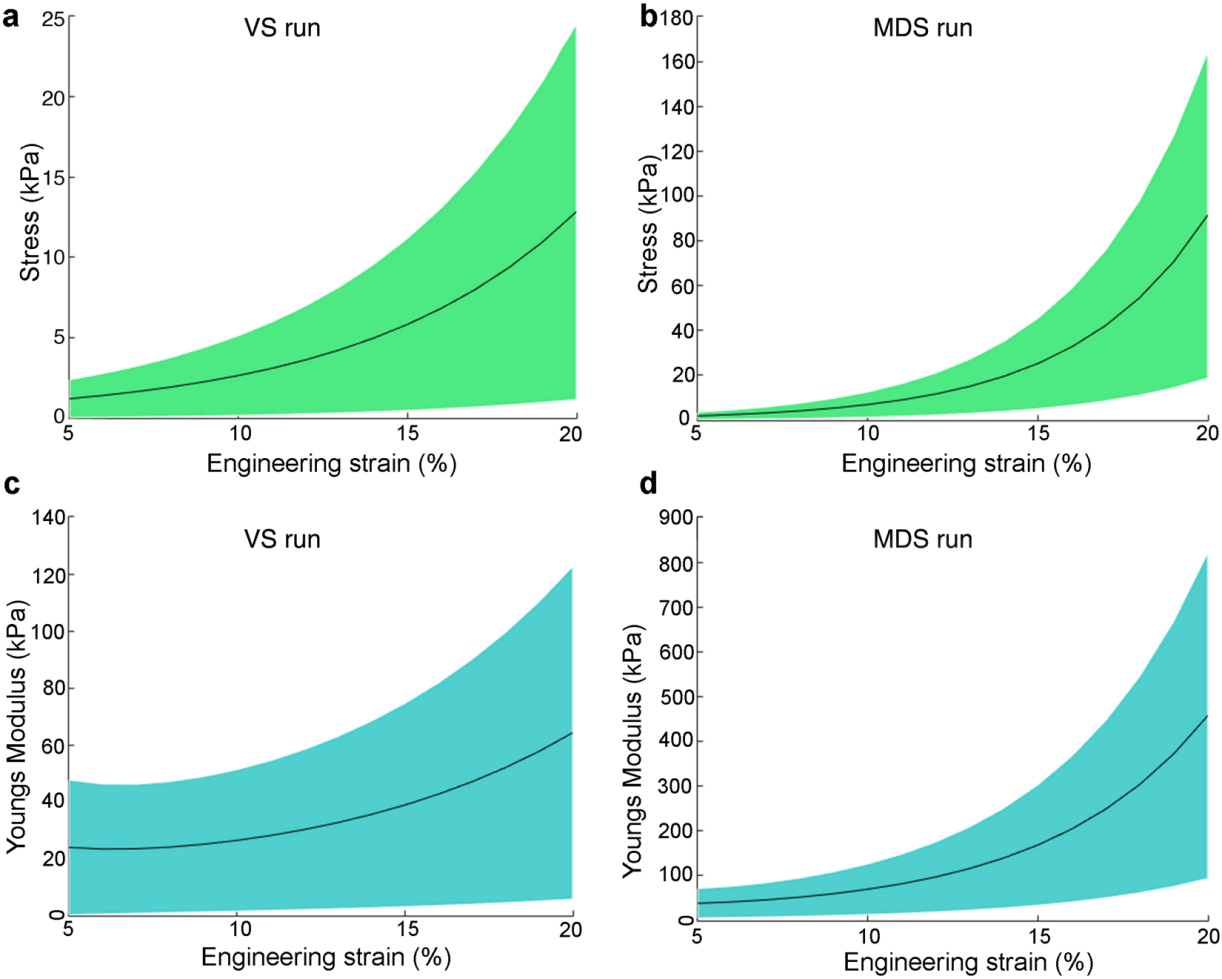
Material properties of the medial labium in zebra finch based on syringeal muscle displacement. (**A**,**B**) Stress-strain relationship of the ML when lengthening the MVM in the (**A**) VS and (**B**) MDS runs (mean (black line) ± S.D. (green). (**C**,**D**) Elastic modulus as function of stress when lengthening the MVM in the (**C**) VS and (**D**) MDS runs (mean (black line) ± S.D. (blue). Mean, S.D. and range values of material properties and curve fitting parameters are listed in Table 1.

To calculate elastic moduli of the ML during VS and MDS actuation runs we combined optical strain and force datasets during the loading curve up to the strain plateau. Stress and elastic modulus increases with strain for both VS (Fig. 6A) and MDS actuation runs (Fig. 6B). Stress in the low strain region, i.e. strain of 5–15% (ref. 26), ranges from 1.0–5.1 kPa and from 1.9–26.8 kPa for VS and MDS runs respectively (Table 1). The elastic modulus in the low strain region ranges from 18.6–34 kPa and from 38.5–178.9 kPa for VS (Fig. 6C) and MDS runs (Fig. 6D), respectively. The different moduli for the two directions is most likely caused by the rod-like LDC that is oriented parallel to **v** **1** and perpendicular to **v** **2**, thus increasing stiffness along **v** **2**.

**Table 1:** Material properties and curve fitting parameters of zebra finch MVM based on VS and MDS runs.

	VS run		MDS run	
	Mean ± S.D.	Range	Mean ± S.D.	Range
ε_max_ [%]	11,9 ± 7,9	6,1–23,2	3,9 ± 4,2	1,2–8,8
σ_1%_ [N/m^2^]	0,6 ± 0,5	0,1–1,1	0,7 ± 0,5	0,3–1,3
σ_5%_ [N/m^2^]	1,0 ± 0,8	0,3–2,0	1,9 ± 1,3	0,7–3,3
σ_15%_ [N/m^2^]	5,1 ± 4,4	1,5–10,5	26,8 ± 17,5	6,6–37,6
σ_max_ [N/m^2^]	2,9 ± 2,4	0,9–6,3	20,1 ± 13,0	5,1–28,85
E_5%_ [kPa]	18,6 ± 17,4	3,1–40,9	38,5 ± 26,5	13,5–66,3
E_15%_ [kPa]	34 ± 29,2	9,7–69,7	178,9 ± 116,8	44,1–250,6
A	0,543 ± 0,490		0,532 ± 0,423	
B	0,157 ± 0,107		0,257 ± 0,132	
r^2^	0,985 ± 0,003		0,969 ± 0,027	

ε_max_ represents strain at maximal displacement, σ_15%_, σ_5%_ and σ_1%_ the tissue stress in at 1, 5 and 15% strain, σ_max_ tissue stress at maximal displacement (ε_max_), and E_5%_ and E_15%_ elastic moduli at 5% and 15% strain. Parameters A and B describe exponential fitting parameters of the stress strain-curves in the function σ = Aexp(B*ε) with the respective regression coefficient R^2^ values. Values for VS and MDS runs are based on measurements on 4 and 3 syrinxes respectively.

The exponential model fit for VS runs is described by:

6 $${\rm{\sigma }}=0,543\,{e}^{0,157{\rm{\varepsilon }}}$$

and for MDS runs:

7 $${\rm{\sigma }}=0,532\,{e}^{0,257{\rm{\varepsilon }}}.$$

Means and standard deviations for the fitted parameters and R^2^ values are listed in Table 1.

### Elastic moduli of mammalian vocal folds

We compiled reported data on elastic moduli in mammalian vocal folds to compare our results (Table 2). For mammalian vocal folds linearity is generally assumed in a low strain region between 5% and 15% (ref. 26). Because we did not observe such linear behaviour in the zebra finch ML in this strain range and the tissue strain did not exceed 11%, we compared elastic moduli across species at 5% strain. In the zebra finch ML, the elastic modulus at 5% strain was 18.6 ± 17.4 kPa for VS and 38.5 ± 26.5 kPa for MDS runs.

**Table 2:** Elastic Modulus values of mammalian vocal folds.

Species	Young’s Modulus	Strain
Zebra Finch MVC(present study)	18,6 kPa	5%
Zebra Finch MDC (present study)	38,5 kPa	5%
Sheep VF^26^	11,7 kPa	5%
Pig IVF^26^	16,3 kPa	5%
Pig SGW^26^	10,2 kPa	5%
Cow VF^26^	29,9 kPa	5%
Dog VF^58^	40,7 kPa	5%
Dog VFb^58^	20,7 kPa	5%
Dog VFc^58^	41,9 kPa	5%
Human TF^38^	30,0 kPa	5%
Lion^48^	11,5 kPa	5%
Bengal Tiger^48^	13,0 kPa	5%
Siberian Tiger^48^	14,0 kPa	5%
Sumatran Tiger^48^	21,0 kPa	5%
Zebra Finch MVC (present study)	760 kPa	40%
Zebra Finch MDC (present study)	39 E + 3 kPa	40%
Siberian Tiger^48^	399 kPa	40%
Brown Rat^22^	~100 kPa	40%
Rhesus Monkey^59^	10369 kPa	40%
Mule Deer^60^	819 kPa	40%
Rocky Mountain Elk^21^	235 kPa	40%
Human^45^	~2000 kPa	40%

Average values of Elastic Modulus as reported in the literature at 5% (low) and 40% (high) strain compared to the corresponding values of zebra finch ML at 5% and 40% strain for MVC and MDC displacements. Please note that the 40% strain values for zebra finches are extrapolated values based on our curve-fitting parameters, but outside of the naturally available range.

### Fundamental frequency predictions

**Figure 7:**
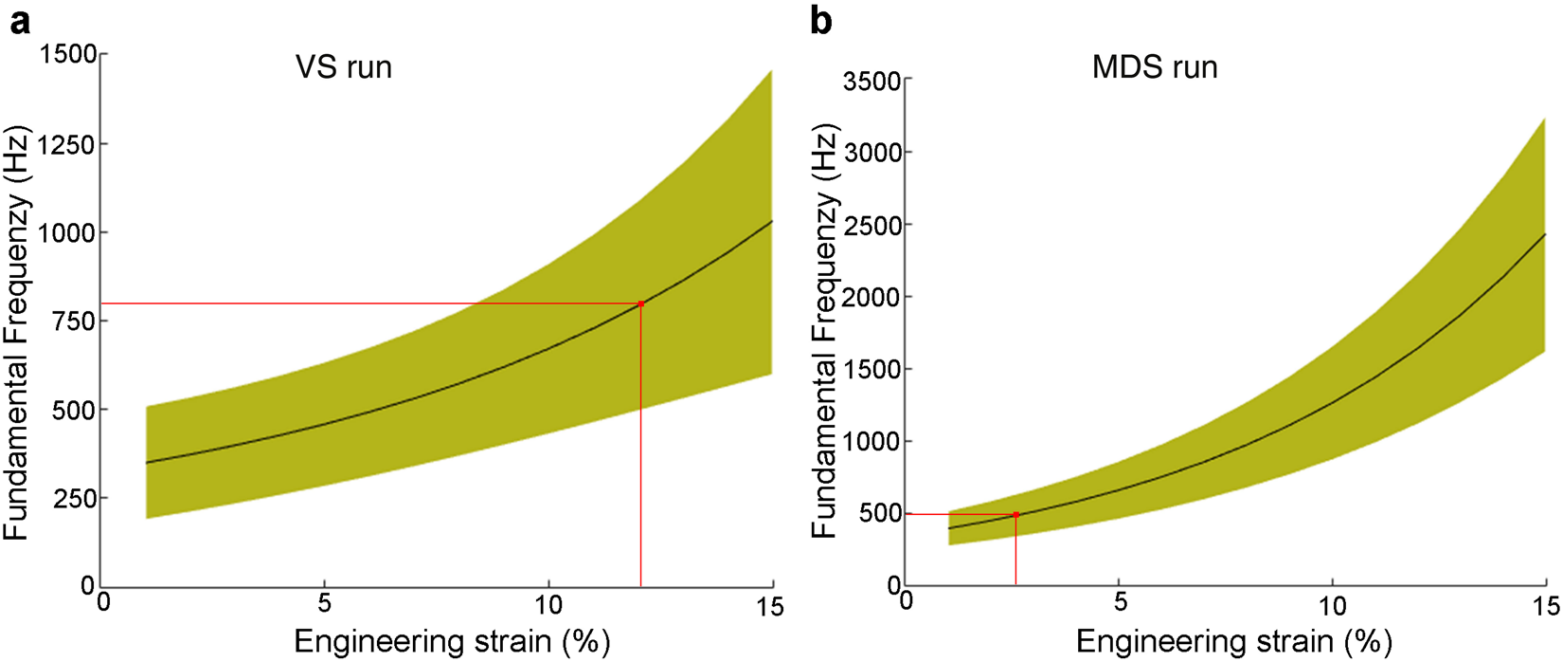
Predicted fundamental frequency range of zebra finch vocalizations based on string model. Fundamental frequency estimates (mean (black line) ± SD (green area)) for (**A**) VS and (**B**) MDS runs. The maximum strain achieved in the ML by modulating *in situ* muscle length is 11.9% and 3.9% for VS and MDS, respectively and indicated by red dots. Thus the predicted range of *f*_0_ modulation is 350–787 Hz with VS and 392–497 Hz with MDS.

We used the measured elastic moduli to predict the fundamental frequency (*f*_0_) with a simple string resonance model (Fig. 7). Predicted *f*_0_ ranged from 350 ± 185 Hz to 787 ± 339 Hz, at 1% and 11.9% strain (plateau) for VS actuation runs and from 392 ± 151 Hz to 497 ± 207 Hz, at 1% and 3.9% strain (plateau) for MDS actuation runs.

## Discussion

We measured 3D deformation of sound producing structures in the excised songbird syrinx as modulated by dynamic forces. We show that the strain in the medial labium (ML) ranges from 0 to 12% and 0 to 4% due to actuation of the VS and MDS muscles respectively. This range is much lower compared to the range available in some mammalian larynges able to lengthen their vocal folds over 40%^40^ and even up to 50% strain^48^. Because the maximal vocal fold strain is very different between species, we compared elastic moduli at a low strain available to all species. At 5% strain the elastic moduli of the zebra finch ML measured 18.6 kPa and 38.5 kPa in the VS and MDS lines of action respectively. These values are within the 10–40 kPa range found in mammalian vocal fold elastic properties including humans (Table 2). The difference in moduli could be caused by anisotropy in the tissue, but this is not supported by fibre composition of the ML in zebra finches^54^. More likely it is caused by the rod-like LDC that may increase stiffness in parallel with the MDS line of action.

As also often observed in elastic modulus data of mammalian VFs^22, 26, 38, 48, 58^ we too observe a rather large variation in the measured elastic moduli between preparations. This variation will in part represent the actual biological variation between the individual tissue properties, but in our measurements additional variation likely originates from our methodology. First, we used an estimate of the ML’s initial cross sectional area based on earlier CT scans and did not quantify the actual preparation, and this cross sectional area may not be constant during the experiments. Furthermore we unfortunately were not able to directly measure the force vector relative to the strain vector, but estimated a maximal angle between the two vectors of 30°. Therefore we overestimate the reported elasticity values with maximally 14%.

The complex procedures and setup needed to calibrate the 3D motion of the MVM did not allow for sound production, because the mirrors prevented the surrounding air sac to be tight. Therefore we used these elastic properties to predict the resonance properties of the tissue and thus the fundamental frequency (*f*_0_) of the sound with a simple string model. Despite the model’s simplicity the predicted *f*_0_ range of 350–800 Hz by VS actuation corresponds surprisingly very well to the range observed in excised syrinx experiments and song. In *ex vivo* experiments the syrinx is in its natural constitution and not affected by muscular pretension or co-activation like *in vivo* ^32^. The minimal *f*_0_ values observed in *ex vivo* recordings by independently controlling bronchial and air sac pressure without muscle activation were 300 Hz^27^, but many preparations produced an *f*_0_ of 500–600 Hz, in correspondence with ML resonance properties of 575 Hz^31^. Minimal *f*_0_ during zebra finch song with sectioned tracheosyringeal nerves, and hence a syrinx without motor activation, was 500 Hz^49^. Thus the predicted minimal *f*_0_ values in zebra finches based on ML elastic properties are in good agreement with observed values.

The predicted maximal *f*_0_ value of 800 Hz at 12% strain due to VS actuation is the same as the highest *f*_0_ observed due to VS stimulation *ex vivo* ^27^. The reported maximum *f*_0_ of song syllables *in vivo* is higher and ranges from 2 kHz^50^ to 5 kHz^37^, predominantly because some individuals include an especially high pitched syllable in their song motif, while other syllables typically range from 500–1500 Hz. The discrepancy between our maximal *f*_0_ prediction based on elastic moduli and *in vivo* observations can be due to several reasons. First, obviously the used string model is a very simplistic 1D estimation of resonance properties and does not include the complex fluid-structure interactions occurring during song. Also, this string model underestimates the frequencies of vocal fold vibrations in the higher strain regions, as the resonance properties do not behave linear in those regions^22, 31^. The non-linear response to higher strains allows for faster oscillations and thus could make up for a higher frequency range. Titze and Hunter^51^ proposed an empirically corrected version for higher strain regions:

8 $${f}_{0}=\frac{1}{2L}\sqrt{\frac{\sigma }{\rho }}[1-0.45\,\mathrm{ln}\,\varepsilon ].$$

Based on this equation, fundamental frequency estimations of the present study range up to 1.5 kHz, which is closer for the observed upper limit of fundamental frequencies *in vivo*.

Second, the high frequency notes observed in zebra finch song could represent a higher mode of oscillation of the entire or partial MVM. It has been proposed that different parts of the MVM can participate in oscillations^31, 32^ altering the effective mass, i.e. the amount of tissue participating in oscillations, but no direct observations have been reported. Additionally, in mammals a layered structure within the vocal folds can affect fundamental frequency prediction as different layers exhibit different viscoelastic properties^26^. Constitutive beam models^52, 53^ account for such layered structure, non-linear response, as well as additional material properties. Different layers based on fibre distribution have also been identified in birds^54^. In zebra finches however, no such layers apart from an epithelium appear to exist^2^. The lack of elastic fibres in the epithelial layer could however have similar effects on the elastic properties as the different anatomy of vocal fold cover and vocal fold body in mammals.

Third, muscle co-activation could results in higher *f*_0_. A co-activation of VS and MDS would lengthen the ML by pulling in opposite directions and increase ML strain perpendicular to the flow. Additionally, MDS shortening also introduces a strain increase along the direction of the flow and how this will affect sound production is unknown. An alternative mechanism could be that MDS shortening lowers the effective mass participating during ML oscillations. Fee^31^ previously also suggested that changes of effective mass could be partially responsible for the high frequency range of zebra finch song. Thus co-activation of VS and MDS could simultaneously increase ML strain and reduce the effective mass, producing a higher resonance frequency than could be achieved by just increasing strain by VS contraction. These hypotheses remain to be tested during sound production.

Our *in situ* paradigm differs in some points from uniaxial stretch experiments, the most commonly used technique in laryngeal studies^22, 26, 38^. Firstly, by keeping the vocal fold *in situ*, we can independently measure forces on the intact system and the isolated vocal fold. Suture stiffness, as well as tissue samples slipping out of the connection clamps have been reported as potential error sources in laryngeal vocal fold studies^39^. Secondly, uniaxial approaches commonly do not measure strain directly at the tissue, but rather derive strain indirectly from the displacement signal^39, 55^. Additionally, uniaxial stretch experiments of laryngeal tissue are most commonly performed in a fluid filled chamber^26, 56^, raising the potential concern of fluid-structure interactions influencing the force measurements. By measuring MVM stress *in situ* directly we circumvent some of the aforementioned errors, like suture stiffness and displacement signal deviation.

Here we did not explore the effects of different dynamical muscle length regimes on ML strain and sound production. Especially using fast muscle actuations complicated dynamic interactions are likely to occur between fluid, structure and acoustics during sound production. Therefore considering only structural forces to predict *f*_0_ as done here may not be accurate. To explore different dynamical regimes of muscle actuation and test the validity to use string models for *f*_0_ prediction under those conditions requires the quantification of acoustic parameters directly, which was not possible with the presented setup.

Interestingly the maximal absolute shortening of the ML reported here is several times lower than the shortening range available to syringeal muscles which allows for faster modulation of ML strain. Syringeal muscle shortening due to electrical stimulation is about 10% of the muscles resting length in ring doves and Bengalese finches^35, 42^. The VS and MDS measure about 4 mm and thus would be able to maximally shorten ~0.4 mm. We currently lack data on how VS shortening bends the flexible MVC and translates the ML in the intact syrinx. However, since we estimated the force vectors of VS and ML strain to be on a maximal angle of 30°, as a rough approximation VS shortening of 0.4 mm would lead to a maximal ML length increase of (0.4*cos(30°) = ) 0.35 mm. However we measured that the ML can lengthen only 180 µm (13% of 1.4 mm), which corresponds to a muscle shortening of 5%. In zebra finches, some syringeal muscles produce maximal positive work at cycle rates of 200 Hz at cyclical strain of 1%^57^. For VS this would correspond to absolute amplitude modulation of 80 µm peak-to-peak at 200 Hz cycling rate, translating to a 70 µm ML length modulation peak-to-peak at 200 Hz cycling rate. Such modulation amplitudes would still allow 40% of the full range of ML strain modulation and thus of *f*_0_ modulation at high cycle rates. The validity of these simple approximations requires further testing, but we propose that operating at low ML strain regimes could aid to allow fast *f*_0_ modulation of vocal output.

The introduced novel methodology allows for the first time to quantify basic biomechanical parameters as syringeal skeleton motion and forces *in vitro* during sound production in birds. The approach will hopefully break ground towards quantifying the acoustic effects of muscle recruitment, and the calibration and testing of sound production models in bird. As such, we will have gained experimental access to the entire neuromechanical control loop of vocal motor control.

## Electronic supplementary material

Supplementary Video S1

Supplementary Video S2

Supplementary Information
