# Supplementary material for: In situ vocal fold properties and pitch prediction by dynamic actuation of the songbird syrinx
Source: Sci Rep. 2017 Sep 12;7:11296. doi: 10.1038/s41598-017-11258-1 (PMC5595934; doi:10.1038/s41598-017-11258-1)
Supplement: Supplementary file 3 — Supplementary Information [file 41598_2017_11258_MOESM3_ESM.pdf]

Supplementary Information

In situ vocal fold properties and pitch prediction by dynamic actuation of the songbird syrinx

Daniel N. Düring[1,2], Benjamin J. Knörlein[3], Coen P.H. Elemans[1,*]

[1] Department of Biology, University of Southern Denmark, Odense, Denmark

[2] Current address: Institute of Neuroinformatics, ETH Zurich and University of Zurich, Zurich, Switzerland

[3] Center for Computation and Visualization, Brown University, Providence RI, USA

*Correspondence to coen@biology.sdu.dk

Supplementary Video S1

3D marker positions and ML strain changes during cyclical VS shortening.

Supplementary Video S2

3D marker positions and ML strain changes during cyclical MDS shortening.
